# Evaluation of Indonesian mangrove *Xylocarpus granatum* leaves ethyl acetate extract as potential anticancer drug

**DOI:** 10.1038/s41598-021-85383-3

**Published:** 2021-03-16

**Authors:** Jason Darmadi, Razethy Rahayu Batubara, Sandiego Himawan, Norma Nur Azizah, Hilyatushalihah Kholis Audah, Ade Arsianti, Evi Kurniawaty, Intan Safinar Ismail, Irmanida Batubara, Kholis Abdurachim Audah

**Affiliations:** 1 Department of Biomedical Engineering, Swiss German University, 15143 Tangerang, Indonesia; 2 Drug Development Research Center, IMERI, University of Indonesia, 10430 Jakarta, Indonesia; 3 Department of Medical Chemistry, University of Indonesia, 10430 Jakarta, Indonesia; 4 Faculty of Medicine, University of Lampung, 35145 Bandar Lampung, Indonesia; 5 Institute of Bioscience, Universiti Putra Malaysia, 43400 Serdang, Malaysia; 6 Biopharmaca Tropica Research Center, IPB University, 16680 Bogor, Indonesia; 7 Department of Chemistry, IPB University, 16680 Bogor, Indonesia; 8 Directorate of Academic Research and Community Service, Swiss German University, 15143 Tangerang, Indonesia

**Keywords:** Drug development, Toxicology, Plant biotechnology, Secondary metabolism, Natural products, Biochemistry, Biological techniques, Biophysics, Biotechnology, Cancer, Cell biology, Chemical biology, Drug discovery, Molecular biology, Plant sciences, Medical research, Molecular medicine, Oncology, Materials science

## Abstract

Local *Xylocarpus granatum* leaves were extracted by ethyl acetate solvent and characterized by TLC fingerprinting and 2D ^1^H NMR spectroscopy to contain phenolic compounds as well as several organic and amino acids as metabolic byproducts, such as succinic acid and acetic acid. Traces of flavonoids and other non-categorized phenolic compounds exhibited intermediate antioxidant activity (antioxidant IC_50_ 84.93 ppm) as well as anticancer activity against HeLa, T47D, and HT-29 cell lines; which the latter being most effective against HT-29 with Fraction 5 contained the strongest activity (anticancer IC_50_ 23.12 ppm). Extracts also behaved as a natural growth factor and nonlethal towards brine shrimps as well as human adipose-derived stem cell hADSC due to antioxidative properties. A stability test was performed to examine how storage conditions factored in bioactivity and phytochemical structure. Extracts were compared with several studies about *X. granatum* leaves extracts to evaluate how ethnogeography and ecosystem factored on biologically active compounds. Further research on anticancer or antioxidant mechanism on cancer cells is needed to determine whether the extract is suitable as a candidate for an anticancer drug.

## Introduction

Indonesia is regarded as one of the richest countries in terms of biodiversity, housing approximately 11% of the world’s flora and fauna^1^. Despite the large quantities of different vascular plants reported in Indonesia, as well as their cultural significance for traditional herbal medicines or bioprospecting program is not yet working properly for Indonesia’s pharmaceutical industry, with 95% of pharmaceutics are imported products^2^. Natural products studied in these herbal plants can be widely used for a large array of medicinal targets, which could transform Indonesia into a powerhouse in biopharmaceutical research and industry.

Natural products are chemicals that are produced and found in nature (plants, fungi, or other living beings). One type of natural product called phytochemicals are derived from secondary metabolites of plants and have been used as medicines since humans began living primarily as food gatherers^3^. Many plants have been known to possess these phytochemical compounds and these compounds are regarded to be potentially profitable as nature-based pharmaceutics. Scientific findings reported that due to their chemical structures, most phytochemicals are biologically active against various types of diseases especially as a potential anticancer drug^4^.

Phytochemicals exhibiting anticancer activity could be used as an alternative for most pure chemo- and radiotherapeutics, as side effects caused by the previously mentioned cancer treatment are still a major concern in the health world^5^. Most phytochemicals are also found to be as effective as and less toxic than pharmaceutical-grade drugs^4^ in terms of treating cancer cells, in this case by behaving as antioxidants which can prompt cytotoxic activity against cancer cells.

Phytochemicals are grouped into several categories depending on their core structure, composition, as well as their functional group. A couple of phytochemical groups such as phenolic compounds and flavonoids are capable of suppressing cancer growth by either inducing mitochondrial or death receptor-mediated apoptotic pathways, by behaving as both anti-aging and antioxidant agents, or by acting as both ways simultaneously^6^. Antioxidant activity is necessary to reduce oxidative stress and free radicals forming within the vicinity of cancer niches^7^. Due to the benefits of antioxidant activity, phytochemicals must exhibit both mentioned biological activities. A type of plant called mangroves possesses these phytochemicals and were recorded both scientifically and culturally in some traditions to be medicinally beneficial^3,8^.

Mangroves are salt-tolerant plants that grow on brackish coasts and seawater systems. Considered to be a cornerstone species, mangroves produce secondary metabolites which helped them thrive on both freshwater and marine systems, as well as provide a habitat for many living beings^3^. In Indonesia, mangroves are a very common sight, as the country hosts the largest area proportion for the mangrove forest ecosystem and 28.5% of the global mangrove population^9^. As mangrove trees are considered to be crucial both culturally and environmentally for many coastal regions; with some locals even utilizing the plants as a staple food, for carpentry, and traditional medicine. By conserving and performing reforestation on their ecosystem throughout Indonesia if not globally, species biodiversity on the surrounding mangrove ecosystems would thrive and locals benefited from the existence of the mangrove forest^2^.

In terms of bioprospecting, mangrove forest conservations would open opportunities in various industrial sectors such as pharmaceutics, cosmetics, and materials. From the conservations, naturally-procured materials such as barks and root can be taken for carpentry, agriculture, and foodstuff; while phytochemicals found in the plants could be analyzed and synthesized in labs for cosmetics and medicinal usage^4^. This would also elevate economic growth for regions containing these mangrove forests if not nationally^9^. Studies have shown that several local mangrove species exhibited antimicrobial activities, as well as containing large traces of bioactive phytochemical compounds^1^; which led the Indonesian government to further support mangrove forest conservation coupled with a national-scale phytochemical extract library project^2^.

A mangrove species called *Xylocarpus granatum* is commonly found throughout South Asia, Northwest Australia, Oceanic islands, and East Africa^10^; with the plant being reported to exhibit cytotoxicity against several carcinoma cells as well as antioxidant activity from its phytochemical content^3,11^. Despite being widespread, studies have found that phytochemicals found in mangroves especially *X. granatum* are different for each geographical site and that the metabolites produced are heavily influenced by their surrounding environment^12,13^. This study was done to examine the contents of Indonesian *X. granatum* species which were understudied phytochemically, with the leaves selected for extraction as there were meager studies conducted globally for *X. granatum* leaves extracts when compared to barks, seeds, and fruits as well as to ease extraction process^8^. This study was also done to give some preview on the difference in phytochemical composition and biological activity of *X. granatum* living in different environments and geographical backgrounds.

## Results and discussion

### Extraction of *X. granatum* leaves extract and phytochemical content analysis by TLC fingerprint and NMR spectroscopy

It was previously reported by Batubara et al. that *X. granatum* leaves simplicia samples contained high traces of tannins, steroids, and saponins^13^. Based on Table 1, the different extracts and control drug Doxorubicin (DOX) were assayed against cervical cancer Hela and breast cancer MCF-7 cell lines to determine which solvent extracted the largest amount of anticancer activity-exhibiting phytochemicals. *X. granatum* leaves extracted in ethyl acetate solvent was found to be more active at inhibiting tumor cells Hela and MCF-7 as well as suspected containing phenolic and antioxidative phytochemicals, such as tannins than when using extracts yielded from water and ethanol. When compared with anticancer drug DOX, the ethyl acetate extract had similar anticancer activity, with the extract shown to be slightly stronger in inhibiting MCF-7 than DOX. It was then suggested that the phytochemical compounds found in *X. granatum* leaves have similar polarity towards ethyl acetate than the other solvents, with some antioxidant-behaving compounds hypothesized to be better extracted by ethyl acetate^14–16^.

**Table 1:** *X. granatum* leaves extracted with different solvents against HeLa and MCF-7.

*X. granatum* leaves solvent extracts	Anticancer activity, inhibition at 500 ppm (%)	
	HeLa	MCF-7
Water extract	64.85 ± 13.26	43.82 ± 9.53
Ethanol extract	85.91 ± 3.23	74.73 ± 2.64
Ethyl acetate extract	92.96 ± 0.12	96.65 ± 0.51
Doxorubicin (DOX)	95.75 ± 0.47	93.95 ± 1.00

One-way Analysis of Variance (ANOVA) was conducted to compare *X.granatum* extract types using water, ethanol, ethyl acetate, and control DOX at 500 ppm cytotoxicity for MCF-7 and HeLa cancer types. Prior to the one-way ANOVA (Table S4 in Supplementary Material), Shapiro–Wilk tests were conducted to test the assumption of normality as reported in Supplementary Material listed as Table S2 and were fulfilled, except for ethyl acetate extract on HeLa (*p* < 0.001). Subsequently, Levene’s tests (Table S3) were conducted to test for assumption of homogeneity and were violated in both cancer types, MCF-7 (*p* = 0.009) and HeLa (*p* = 0.006). Due to this violation, a Brown-Forsythe (BF) F test was conducted (Table S5), and the tests were significant for both samples. The test resulted in F (3, 2.363) = 71.884, *p* = 0.007 for MCF-7 and F (3, 2.242) = 12.571, *p* = 0.060 for HeLa.

Games-Howell posthoc tests were also conducted (Table S6), with pairwise comparisons of extracts based on cancer type. The pairwise comparisons in MCF-7 showed that ethyl acetate extract (*M* = 96.65) was significantly higher than water (*M* = 43.82, *p* = 0.026), ethanol (*M* = 74.73, *p* = 0.010), but significantly lower than DOX (*M* = 93.95, *p* = 0.074). However, pairwise comparisons in HeLa showed that ethyl acetate extract (*M* = 92.96) was also significantly lower than DOX (*M* = 95.75, *p* = 0.018), but non-significantly higher than water (*M* = 64.85, *p* = *0.1*60) and ethanol (*M* = 85.91, *p* = 0.152), with ethanol significantly lower than DOX.

Results of the test could be explained due to the small sample size (*n* = 3) and outliers for every category of extract type across cancer type. The BF test is based on the application of ANOVA using deviations from the median instead of the mean, hence it is not as easily affected by outliers as the standard ANOVA test^17^. It is considered a robust test, controlling Type I error for various data shapes and sizes^18^, with one of the highest statistical power values out of 8 other variance tests. Although its power decreases with smaller group sizes (*n* = 5)^19^. Based on the statistical results and phytochemical screening, it was decided that *X. granatum* leaves ethyl acetate extract would be studied as the main extract.

**Figure 1:**
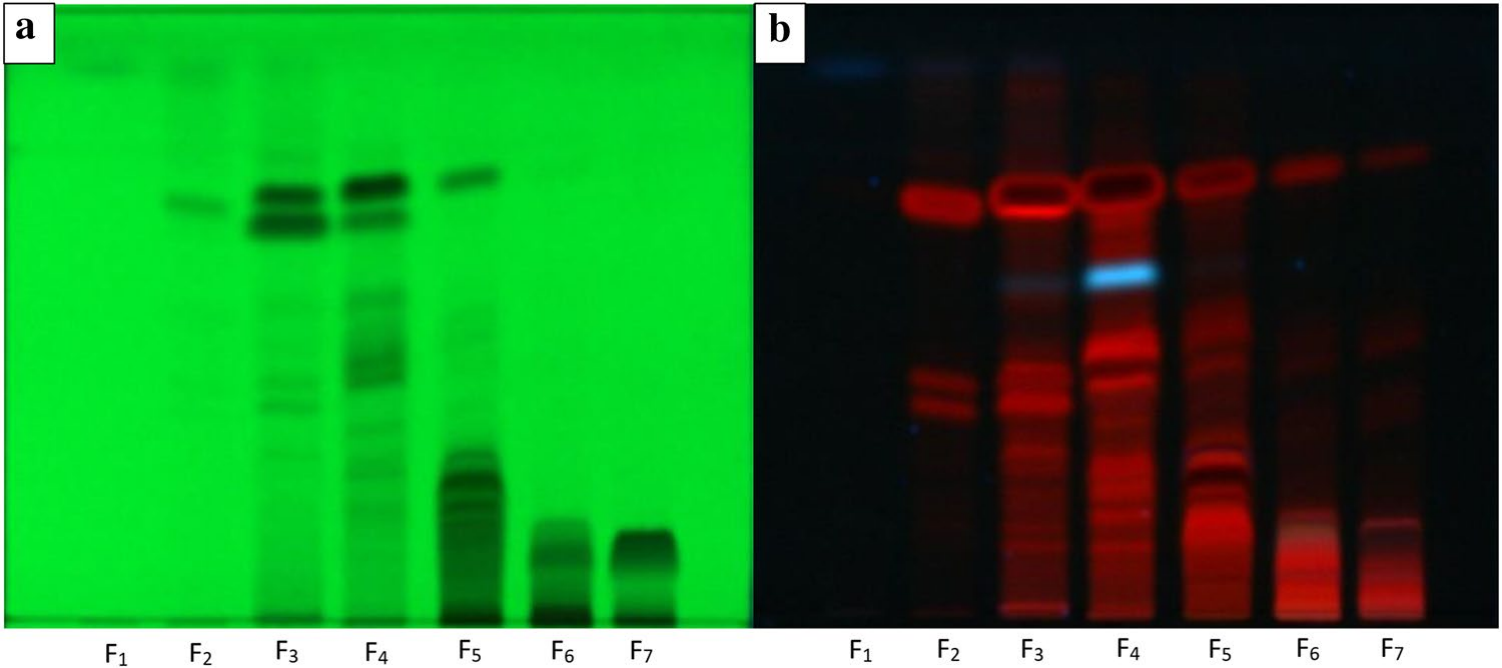
TLC Chromatogram from *Xylocarpus granatum* leaves ethyl acetate extract fraction 1 to fraction 7 (F_1_ to F_7_) using mobile phase chloroform:dichloromethane 9:1 (v/v) with (**a**) UV exposure at 254 nm and (**b**) 366 nm.

The ethyl acetate extracts were fractionized into seven fractions by TLC extract fingerprinting and were visualized by different wavelengths for comparison as seen in Fig. 1a,b. Chromatogram observations of 254 nm (Fig. 1a) and 366 nm (Fig. 1b) showed numerous bands being visualized differently in terms of intensity and amount when compared to one another. The TLC plate was exposed to wavelength 366 nm, different colored bands could also be seen, with each color corresponding to a certain flavonoidic group of phytochemical compound. The blue colored-band represented flavonoids, flavonons, or flavonols; while red-colored bands often represented anthocyanidin-related compounds (Fig. 1b)^20^.

Similarities in band intensity and patterns could be seen in Fig. 1a,b, with bands seen more clearly in the TLC plate exposed at 366 nm compared with 254 nm which only had several contrasting monochromatic bands. The purpose of observing in two different wavelengths was to determine which wavelength was better suited for visualizing extract bioautography, in this case, phytochemicals in *X. granatum* leaves ethyl acetate extract. Phytochemicals behaved differently in different wavelengths due to their structure and could go undetected in certain wavelengths (Fig. 1a,b). Both wavelengths are necessary for rechecking purposes, with intensity of several bands in 254 nm had more clarity whereas color diversity could be seen in 366 nm.

**Figure 2:**
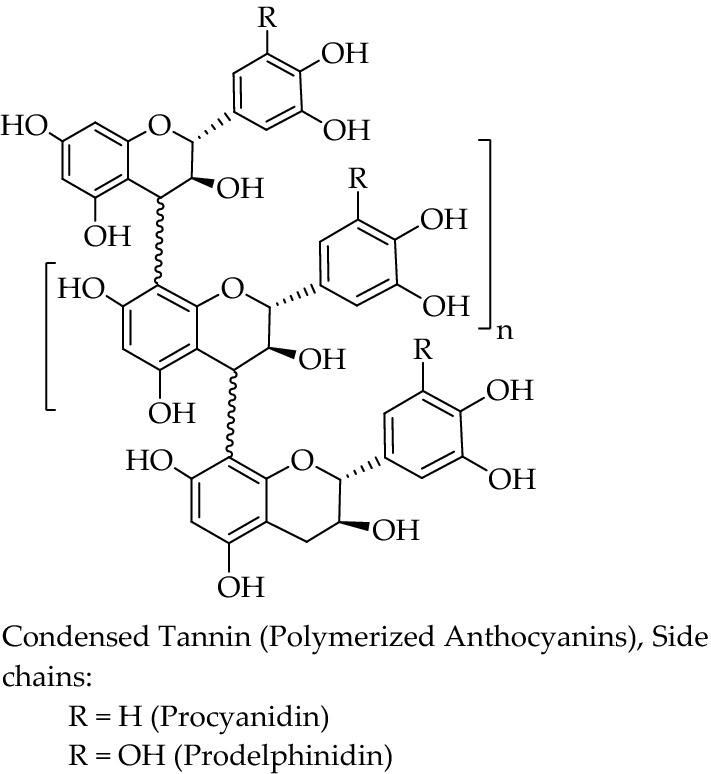
Chemical structure of condensed tannin made from flavonoid derivatives.

Based on Fig. 1b, there were many phytochemicals found throughout the fractions which might indicate the presence of anthocyanidin-like compounds with several fractions showing traces of other flavonoidic groups. The red bands found in Fig. 1b were hypothesized to be formed mostly by condensed tannins made out of various anthocyanidins or anthocyanins (Fig. 2); while the blue traces might have been flavanols or in this case flavan-3-ols separated from some condensed tannins found in the extract^21^. The polymerized tannins in extract might have a slightly unstable structure due to their weak inter-flavonoidic bonds and might have a tendency to degrade into several flavonoid classes. While this might also mean that both saponins and steroids do not have a high affinity towards ethyl acetate as they were not seen in TLC bands and might be present in the other solvents which were not as anticancerous as the flavonoid-like compounds found in the ethyl acetate extract; further thorough investigations on metabolites profiling is highly recommended to determine the exact active compounds involved in the anticancer activity of ethyl acetate extract as well as other solvents whether in TLC or other analytical methods.

Qualitative phytochemical analysis was performed for flavonoids, tannins, and saponin contents on ethyl acetate extract to check on the TLC fingerprinting results. It was found out that by using the Froth test, ferric chloride test, and Willstatter’s flavonoid screening test^13,14^ that qualitatively there were no traces of saponins and tannins, with indication of flavonoids present. This confirmed that there were no saponins found in the ethyl acetate extract and that most tannins found in the simplicia were suggested to degrade into various forms of flavonoids as the flavonoids were not detected in the first simplicia samples.

**Figure 3:**
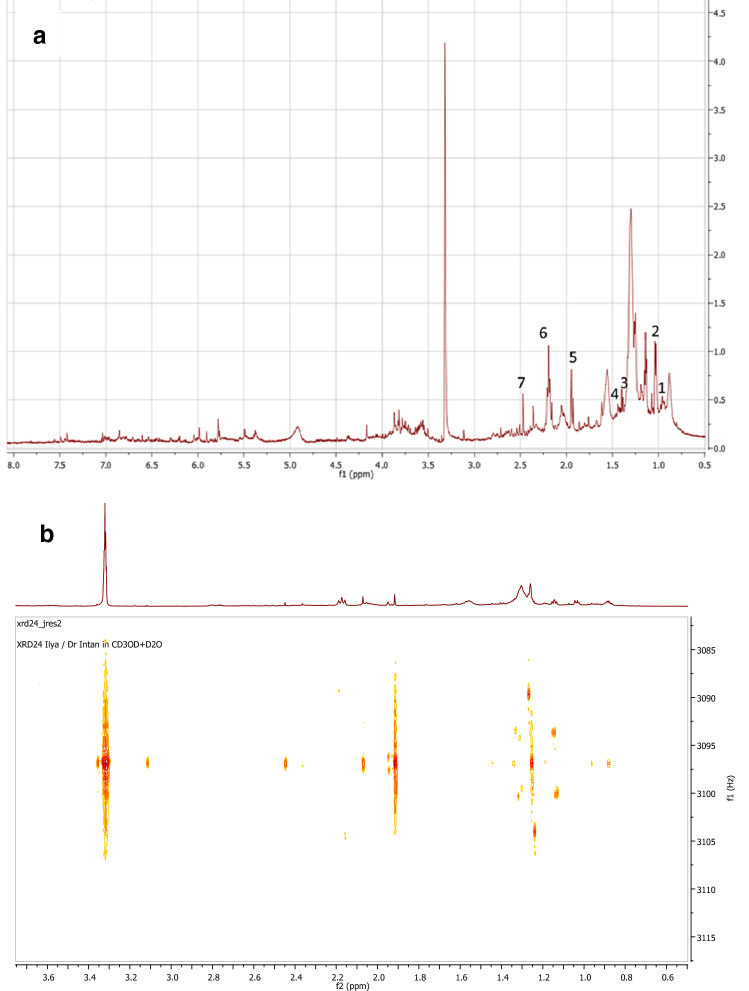
NMR Spectroscopy result from *X. granatum* ethyl acetate extract. (**a**) 1D ^1^H NMR (CD_3_OD + D_2_O, 500 MHz) *δ*0.94 (d, J = 7.0 Hz, isoleucine), 1.03 (d, J = 6.1 Hz, valine), 1.39 (d, J = 6.7 Hz, lactic acid), 1.46 (d, J = 6.7 Hz, alanine), 1.93 (s, acetic acid), 2.20 (q, J = 7.5 Hz, propionic acid), 2.47 (s, succinic acid), 4.07 (q, J = 7.0 Hz, lactic acid). (**b**) 2D ^1^H NMR (CD_3_OD + D_2_O, 500 MHz) *δ*7.02 (d, J = 0.8 Hz), 3.11 (s), 2.46 (s, succinic acid), 2.07 (s), 1.95 (d, J = 1.4 Hz), 1.93 (s, acetic acid), 1.33 (d, J = 5.4 Hz), 1.31 (d, J = 7.0 Hz), 1.26 (t, J = 14.6 Hz), 1.14 (d, J = 5.7 Hz).

To further analyze the composition of the ethyl acetate extracts, analysis was performed by proton nuclear magnetic resonance (^1^H NMR) spectroscopy. By analyzing the spectral data groupings in the extract, it was found out that most spectrums corresponded to certain metabolites, with each associated compound stood for a single grouped NMR spectral bin (Fig. 3a). Most of the compounds visualized were both metabolites and metabolic byproducts behaving as leaf exudates, including amino acids, several acids such as lactic acid and propionic acid, as well as carboxylic groups which might be formed by ethyl acetate solvent degradation such as acetic acid (Fig. 3a); while small traces of phytochemical groups in Fig. 1b were found. Two-dimensional (2D) measurement of ^1^H NMR spectroscopy was also performed to further characterize the composition of the extracts. The results showed that two metabolites had been fully analyzed which are: succinic acid (δ2.46,s) and acetic acid (δ1.93,s) (Fig. 3b). These results corresponded with the NMR analysis visualized in Fig. 3a. However, this result showed that compounds found in the extracts were mostly metabolites and byproducts in large quantities which might have attenuated the visualization of other bioactive compounds.

The metabolites and byproducts visualized in Fig. 3a,b could be used as a medicinal extract as the exudates were reported in studies to be biologically active and used by plants as a defense mechanism (Table 2), but due to the extract’s highly crude content, the anticancer activity assayed in Table 1 might be an attenuated result and higher inhibition might be possible if the *X. granatum* leaves ethyl acetate extract could be further fractionized.

**Table 2:** Compounds found in leaves ethyl acetate extract after analysed by ^1^H NMR spectroscopy.

Metabolic compounds	Medicinal usage	Reference
Isoleucine	Antihepatic encephalopathy, anticatabolic, and antitardive dyskinesia activity for branched-chain amino acid	^22^
Valine	Muscle stimulant and growth activity for branched-chain amino acid	^23^
Alanine	Protein synthesis and sugar metabolism	^24^
Acetic Acid	Antibacterial and antifungal activity	^25^
Lactic Acid	Antioxidant and alkalizing activity	^26^
Propionic Acid	Antifungal activity	^27^
Succinic Acid	Antiallergenic activity	^28^

It was also possible that almost all metabolites heavily visualized in the NMR data were some type of sub-structure degraded from other bioactive phytochemicals, for instance: propionic acid and lactic acid (Table 2) could have been degraded from commonly found *X. granatum*-based semipolar limonoids such as xyloccensins, xylogranatins, and gedunins which were reported to have anticancer activity towards several cell lines^29,30^. A new type of specific anticancer compound might also be present in the ethyl acetate extract if analysis was performed at a structural and molecular level. For now, however, the organic acids and amino acids were only used for preliminary phytochemical fingerprinting as the evidence of any significant specific compounds were lacking and further analysis on metabolomics should be done.

Liquid chromatography-mass spectrometry (LC–MS/MS) was recommended to be the next characterization step to validate the results from NMR spectroscopy and TLC fingerprinting for metabolic fingerprints of the ethyl acetate extracts. However, due to time constraints and logistical difficulties at the time of writing, the chemical composition of ethyl acetate leaves extracts for *X. granatum* were only characterized by simple TLC as well as NMR. While the extracts were indicated to contain possible biological activity-behaving phytochemicals, the exact phytochemical groups hypothesized are still inconclusive and need further investigation such as LC–MS/MS to finally determine which specific compounds exhibit anticancer activity. For now, as traces of flavonoids were found from preliminary phytochemical screening, total phenolic and flavonoid content of extracts were tested to determine how many percentages of flavonoids had yielded in the extracts as a possible anti-cancer drug as well as antioxidative compound.

### Antioxidant activity of *X. granatum* leaves ethyl acetate extract

Most phenolic compounds, particularly flavonoids, develop antioxidant activity by having aromatic structures that could help the compound to retain stability after scavenged by free radicals found on cells. Several types of functional groups are also capable of improving or suppressing antioxidant activity, for instance: hydroxyl groups (-OH) depending on their amount and their placement in the aromatic ring could interfere with electron transfer in the compound due to their hydrophilicity; carbonyl groups (C=O) and double bonds in aromatics could also improve resonance effect of compound; methoxy groups (–OMe) could either suppress or improve antioxidant activity by increasing hydrophobicity and structure planarity; and glycosylation works similarly to methoxylation by steric hindrance or polarity^6^. When in proximity, free radicals would scavenge proton ion [H +] from phytochemicals and the compounds would then regain structural balance by donating electrons from their own aromatic structure or oxygen-based functional groups towards the scavenged site^31^. Due to this principle, antioxidant activity is deemed as necessary for anticancer agents to bind with reactive radical oxygen species (ROS) or radical nitrogen species (RNS) produced near cancer sites. Another usage of antioxidants as anticancer drugs is by triggering cancer cell apoptosis^32^, although some aspects of its full mechanism are still unknown and have only been proposed^33^.

*X. granatum* leaves ethyl acetate extracts were tested for total phenolic content (TPC) and flavonoid content (FC) to determine how much were contained and still functional as an antioxidant agent. It was found out that 28.36 ± 0.50 ppm of TPC were contained in the extract, with 85.75% (24.32 ± 1.19 ppm) of the polyphenols being flavonoids. Extract was also assayed for antioxidant activity by 2,2-diphenyl-1-picrylhydrazyl (DPPH) radical scavenging assay and it was found out that antioxidant DPPH IC_50_ of extract was approximately at 85 ppm, as seen in Table 3. This meant that the extract was labeled as having an intermediate antioxidant activity, despite still being not as effective as ascorbic acid which was regarded as a very strong antioxidant^15^.

**Table 3:** Antioxidant IC_50_ for extract and ascorbic acid.

Extract sample	Antioxidant Activity DPPH IC_50_ (ppm)
Ascorbic Acid	7.24 ± 0.39
*X. granatum*	84.93 ± 12.93

Flavonoids are categorized in a larger group of phytochemicals called phenolic compounds, which exhibit most of the antioxidant properties of an extract. Most of the antioxidant activity found in the extract was suggested to be exhibited by flavonoids and other phenolic acids as well as polyphenols, as traces of said phytochemicals were found prominently in the extract. Despite this, the mode of action and mechanism for reported flavonoids on cancer cells and antioxidants were not yet known but it was speculated to be linked with their structure and antioxidant behavior. Studies have shown that antioxidative flavonoids could interact with cell membranes by aggregating in their membranes or latching to other peptide or glycosylated compounds to pass transport proteins. Flavonoids could form ion-complexes with metal ligands from their scavenging and metal-chelating properties, which made delivery to cells easier^34^. Aggregation of flavonoids or other polyphenols was due to their lipophilic properties likely from their –OH and glycosidic groups, forming lipid rafts and shells to help endocytosis^35^.

While the lipophilicity of flavonoids affects cell membranes, there are studies that have shown that the flavonoidic effects are related to multiple properties of the lipid bilayer itself and may indirectly help as an initiator of anticancer action. In one study, due to the accumulation of quercetins inside the bilayers, cell membrane conductivity in rat distal colon epithelium were disrupted which caused an inability to store Ca^2+^ inside cells. Another study had also reported that some flavonoids such as apigenin and genistein which contained several –OH groups could increase the rigidity of both hydrophobic as well as hydrophilic sites of the bilayer, which in turn cascaded into activation of membrane enzymes and receptors that synergize with the anticancer action of the flavonoids. This was also supported by a study that reported findings where several hydroxyl-containing flavonols could influence the activity of membrane transport proteins known as P-gp and MRP1 which are a major contributor to multiple drug resistance (MDR) properties as well as repression of apoptotic pathways in cancer cells. By modulating the fluidity of membrane lipids, these proteins were inactivated and reversed MDR, thereby indirectly weakening the mitochondria of HCT-15 colon cells which prompted programmed cell death by mitochondrial lysis^35^.

The flavonoids found in the extract were suspected of containing several functional groups, in particular some double bonds in C_2_=C_3_, several –OH groups, as well as C_4_=O^6^ in its structure aside from exactly having some aromatic rings. When B-ring was oxidized by radicals due to catechol or –OH in 3′ and 5′, the structure became less stable. The double bond and also ketone in C-Ring served as an electron donor for B-ring which would maintain a stable structure^31^. Even though double bonds in C-ring might exist in the flavonoids from ethyl acetate extract, both A- and B-rings most certainly had aromatic nuclei, which would give a stable structure by resonance effect of electrons even after oxidized twice^36^. Other less significant antioxidant promoters which could be present include methoxylation in certain rings and also C-or O-glycosides^6^. Further tests, fractionation, and structural analysis should be done to prove whether the aforementioned functional groups exist in the extract and whether the flavonoids were the primary antioxidant phytochemicals of the extract.

Despite the intermediate antioxidant activity found in the ethyl acetate extract and their anticancer activity^6^, studies have also found that a high intake of phenolic compounds especially low-soluble flavonoids and tannins could induce acute toxicity^33,37^. Reports also showed that some antioxidative compounds may also induce pro-oxidant effects such as in several oxidized phenol B ring flavonoids (apigenin and kaempferol) which were involved in mitochondrial respiration and erythrocyte hemolysis^35^ as well as simpler phenolic compounds like ascorbic acid which could increase atherosclerosis risks. While in large concentration ascorbic acid and many phenolic compounds are antioxidants and co-antioxidant, these compounds could turn pro-oxidant when used in low concentration or when interacting with free metal ligands such as iron ions due to their inherent reactivity to these compounds^38^. Due to this, it was necessary to test whether the phytochemicals found in the extracts that promote anticancer activity did not also behave as a cytotoxic towards normal cells.

### Toxicity properties of *X. granatum* leaves ethyl acetate extract

As the purpose of the extract is to be used by humans to treat cancer and also to be an antioxidant agent, the extract should not be toxic to humans. One way to test the toxicity of a chemical is by exposing it to a simple model organism such as brine shrimp *Artemia salina*. As some of the phytochemical compounds of the extracts had some indication of being degraded, the resulting compounds should be tested for brine shrimp lethality test (BSLT) to determine whether the altered chemicals became more toxic or not. The result was interpreted and analyzed extract concentration when *A. salina* lethality reaching 50% or LC_50_. All brine shrimps seeded in ethyl acetate extract were found to still be alive, which gave LC_50_ of extract above 1500 ppm. This suggested that the antioxidative phytochemicals within the extracts were not lethal towards brine shrimps^39^.

**Figure 4:**
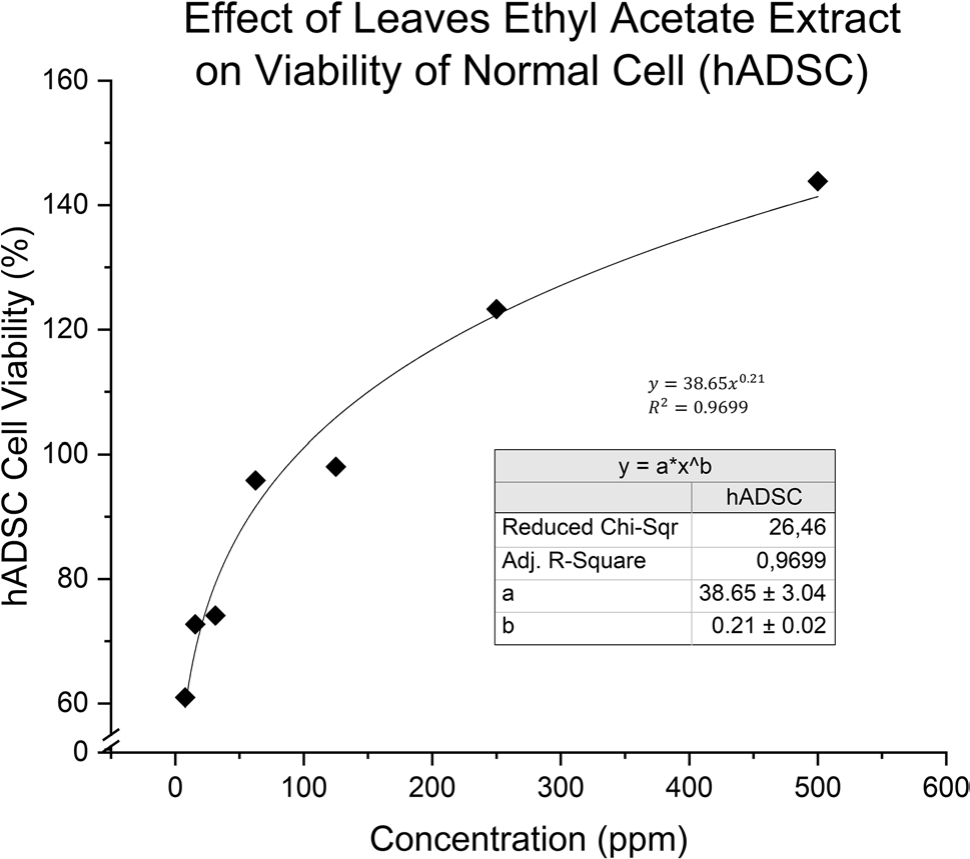
Effect of *X. granatum* towards viability of human adipose-derived stem cell (hADSC).

The extract was also examined for compatibility towards normal human cells, as BSLT only showed how toxic a substance is to a simple model organism and not a complex one such as a human cell. Ethyl acetate extract was assayed using 3-(4,5-dimethylthiazol-2-yl)-2,5-diphenyl tetrazolium bromide (MTT) cytotoxicity assay for cell viability against human adipose-derived stem cell (normal cell hADSC) with comparison to anticancer drug Cisplatin (CDDP). Based on Fig. 4, the viability of hADSC increased when exposed to higher concentrations of extract, with cell growth exceeded 100% after exposure to concentrations higher than 78 ppm. The results indicated that the phytochemicals contained in the extract behaved similarly to growth factors or as stem cell activators. Flavonoids and other phenolic compounds found in extract might behave as enzyme inhibitors or stimulators in growth factor pathways such as VEGF or TGF-β similar to the findings by Arudina et al., where mangosteen skin extract was found to enhance mesenchymal stem cell MSC proliferation to up to 180% by antioxidant activity which stimulated the formation of FGF-2^40^.

**Figure 5:**
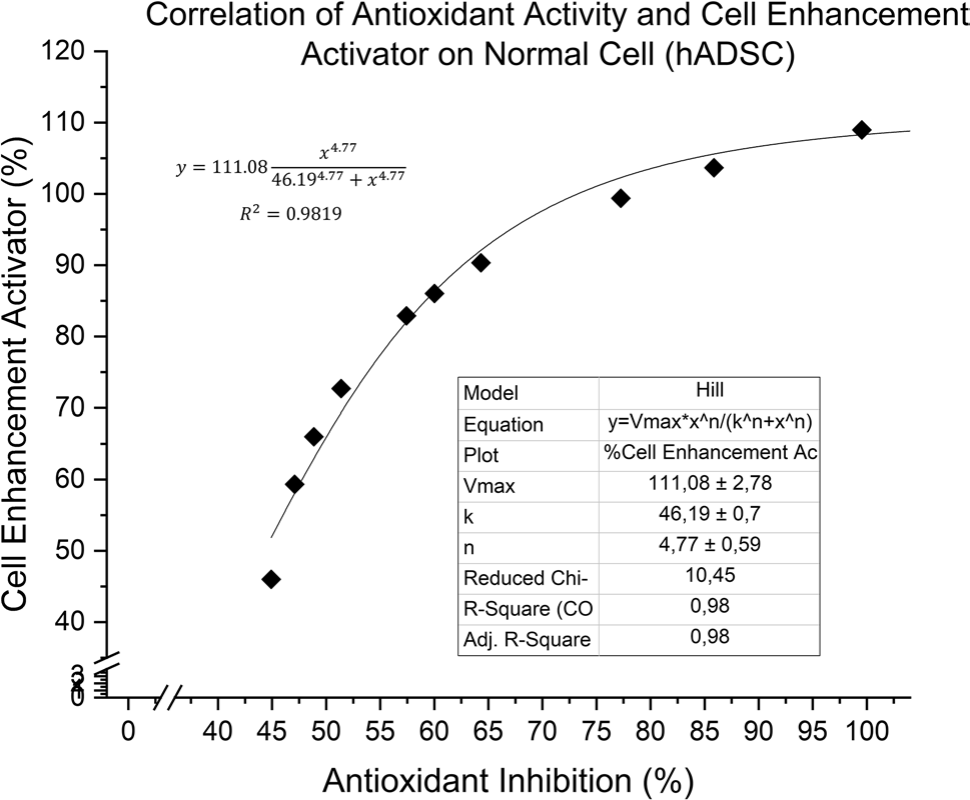
Correlation between antioxidant activity and cell enhancement activator of extract towards hADSC.

To determine whether the antioxidant activity of phytochemicals correlates to cell growth activation, the antioxidant activity of the extract was plotted against cell viability or enhancement activator as seen in Fig. 5. Using Hill’s Sigmoidal Model only for curve fitting, the plot showed high R^2^ at + 0.98, with cell viability slowly increasing after surpassing 109% while antioxidant activity stopped at 100%. This suggested that the antioxidative flavonoids found in the extracts contributed to life-prolonging properties as well as stem cell proliferation activity with its mechanism hypothesized to be similar to some enzyme inhibitors or allosteric ligands such as growth factor FGF-2 which could bind to receptors for signal proliferation and in cell growth pathways^41,42^.

Aside from triggering activation of cell growth pathways, antioxidants in the extracts could also promote anti-aging activity by reducing oxidative stresses in cells, as accumulation of genetic damages from ROS could cause cell senescence and mutations^43^. Several plants have been identified to contain anti-aging substances and these substances were also directly linked with antioxidants. For instance, *X. granatum* bark extract was reported to reduce acne at DPPH IC_50_ 24 ppm and was considered as a potential anti-aging and skin cosmetics agent^44,45^. FC found in the *X. granatum* leaves ethyl acetate extracts could promote lifespan increase for normal cells by attacking ROS while also regulating pathways for several growth hormones such as IGF and VEGF^42^.

Despite the overall ability of inhibiting tumor cells, natural growth factor, and anti-aging activity of the phytochemicals; *X. granatum* leaves extract might also fully suppress cell cycle arrest and apoptosis pathways on nearby cells or stem cells, causing new cancer stem cells niches to form and increase tumorigenesis as well as cancer proliferation^46^. Further research is necessary to determine whether the long-term usage of extracts would cause the emergence of drug-resistant cancer cells.

### Anticancer activity of *X. granatum* leaves ethyl acetate extract

As the extract was found to be capable of inhibiting HeLa and MCF-7, tests were performed by using MTT cytotoxicity assay to determine extract efficacy compared with CDDP against several cell lines. Cell lines chosen were regarded as significantly prevalent for Indonesian cancer cases^47^, which are: breast cancer T47D, colorectal cancer HT-29, and HeLa. Results were shown as extract concentration when cancer proliferation and growth inhibition reached 50% or anticancer IC_50_. DMSO 2% was used as control negative and was found to be non-cytotoxic towards cancer cells and stem cell, with absorbance level on par with the cells treated with the lowest concentration of *X. granatum* leaves ethyl acetate extract in solvent DMSO 2%.

Visual comparison for anticancer activity against HT-29, HeLa, T47D for *X. granatum* leaves ethyl acetate extract and CDDP can be seen in Fig. S1, S2, and S3 respectively as listed in the Supplementary Material. Anticancer activity was found to be present in extract with the highest potency reached against colorectal cancer HT-29 (Table 4), with extract anticancer IC_50_ being even lower than CDDP due to the intended use of CDDP was only against cervical and breast cancers. This suggested that the extracts had was successfully delivered to both hADSC and cancer cell lines due to the antioxidant compound’s structures which was said to be lipophilic^34,35^. The anticancer activity towards colorectal cancer, however, was not significantly stronger than CDDP. This was due to CDDP being a pharmaceutical-grade single compound alkylating agent that was even cytotoxic towards hADSC, while the extract used was still crudely extracted. Anticancer activity of extract was attributed to the phenolic configuration and side groups of flavonoids, similar to its antioxidant activity^6,32,48^; yet not much was known for its exact mechanism.

**Table 4:** Anticancer IC_50_ for extract and CDDP against cells.

Sample	Anticancer MTT cytotoxicty IC_50_ (ppm)			
	hADSC	HT-29	HeLa	T47D
*X. granatum*	Activator (> 73.8) Inhibitor (< 73.8)	42.50 ± 36.56	559.57 ± 857.79	77.76 ± 66.70
Cisplatin (CDDP)	12.68 ± 4.59	115.91 ± 32.47	1.86 ± 1.38	31.08 ± 13.95

One-way ANOVA tests were conducted for the calculated IC_50_ of the *X. granatum* and CDDP samples respectively, across all Cell Line Types (Table S9 listed in Supplementary Material). Before the one-way ANOVA, Shapiro–Wilk tests were conducted to test the assumption of normality across eight independent categories —*X. granatum* and CDDP with hADSC, HeLa, T47D, and HT-29 respectively were fulfilled, except for the CDDP with HeLa category (*p* = 0.001), as seen in Table S7 in Supplementary Material. Subsequently, Levene’s tests (Table S8) were conducted and assumptions of homogeneity based on mean were violated for IC_50_ of *X. granatum* (*p* = 0.002) and CDDP (*p* = 0.001). Due to this violation BF test was conducted (Table S10), however the tests were non-significant for both samples. The test resulted in F(3, 2.032) = 1.092, *p* = 0.509 for *X. granatum* and F(3, 2.003) = 0.931, *p* = 0.555 for CDDP.

Similar to results from comparing *X. granatum* extract types across HeLa and MCF-7, results of this test could also be explained due to the small sample size (n = 3) and outliers for every category of Sample and Cell Type. The BF test is based on the application of ANOVA using deviations from the median instead of the mean^14^, hence it is not as easily affected by outliers as the standard ANOVA test. It is considered a robust test, controlling Type I error for various data shapes and sizes^15^ with one of the highest statistical power values out of 8 other variance tests^16^. However, it should be noted that the BF test statistical power decreases with smaller group sizes (n = 5).While the results showed that there are statistically not significant differences across independent categories for both *X. granatum* comparative tests, extracts had indeed showed some potential as anticancer drug as ethyl acetate extracts could inhibit HT-29 cell lines with stronger efficacy than CDDP.

**Figure 6:**
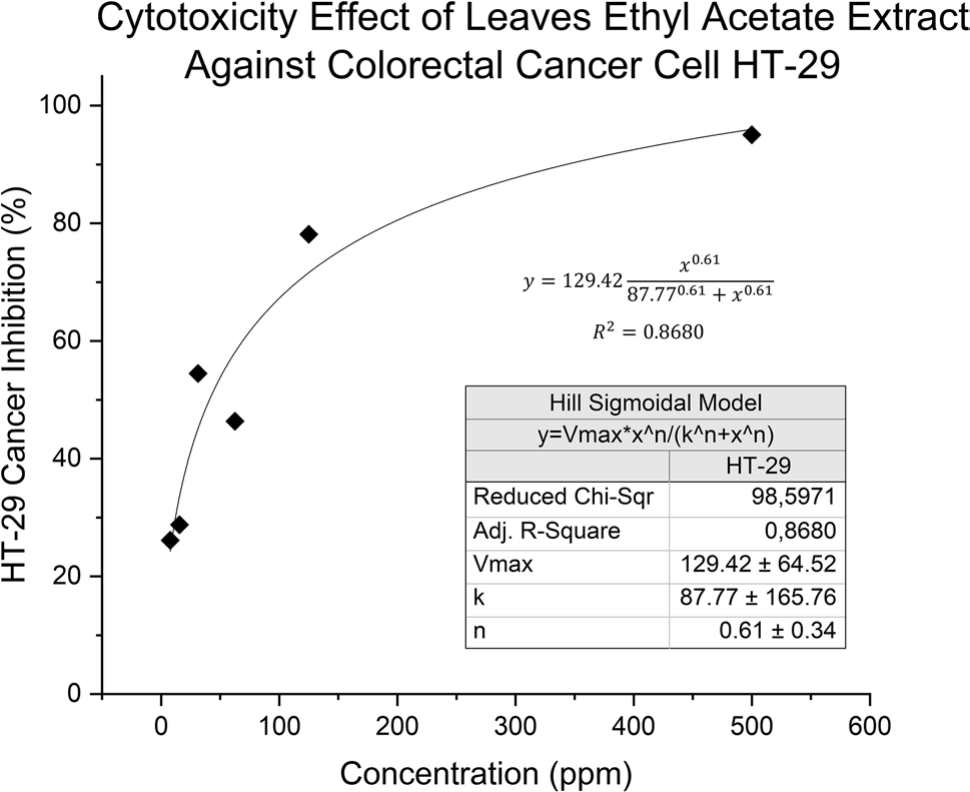
Cytotoxicity effect of *X. granatum* against of HT-29.

One possible way for the phytochemicals in the extract to effectively inhibit cancer cells, in this case against HT-29, would be by quenching the enzymes corresponding to cell growth and proliferation as suggested in Fig. 6. Based on the figure, the extract had a similar activity trend (Hill sigmoidal model fitting) to an enzyme–substrate or inhibitor. However, despite the *Vmax* and *Km* were defined in the curve, this did not mean that the Michaelis–Menten kinetic definition could be used for the formula and curve. Figure 6 only suggested that the extract would inhibit HT-29 growth as though a phytochemical substrate would bound to enzymes that regulated cancer cell growth.

The HT-29 inhibition kinetics might suggest that the chemicals could bind to some allosteric enzymes in cancer cells which would suppress and even inhibit activity of the enzyme, similar to the indicated mechanism of natural growth factor towards hADSC. One study had shown that myricetin-like flavonoids contained in a certain extract were able to suppress a biomarker enzyme for HT-29 called hFEN1 which non-competitively inhibits cancer proliferation, rendering DNA repair and replication at certain genes useless^49^. Flavones flavonoids could also regulate transport proteins such as MCT-1 to increase lactate uptake into the mitochondria of HT-29, causing oxidative stress and triggering apoptosis^50^. These mechanisms are plausible but need further research on structure-assisted relationship (SAR) of fractionized phytochemicals towards both biomarkers.

**Figure 7:**
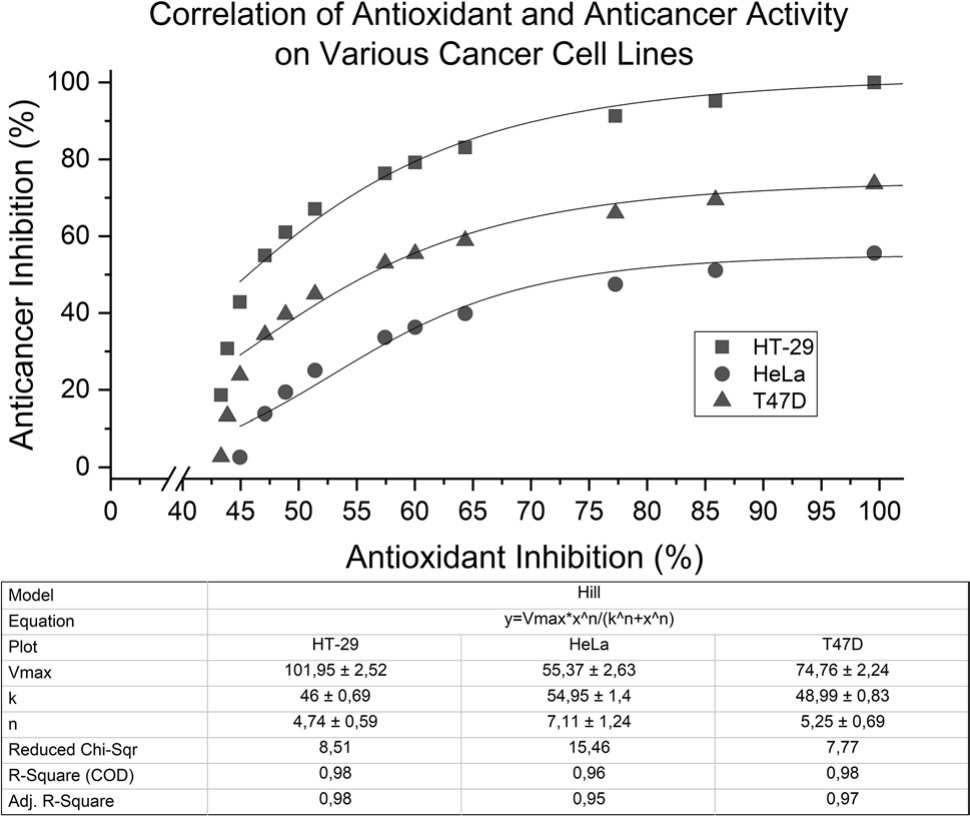
Correlation between antioxidant activity and cell anticancer activity of extract towards various cancer cell lines.

When anticancer and antioxidant activities of the extract were plotted for correlation to determine whether TPCs were responsible for anticancer activity against HT-29, HeLa, and T47D; the R^2^ for all fittings were significantly sigmoidal at around + 0.97 (Fig. 7  and Table S1). Similar to correlation plot for Fig. 5, Hill’s sigmoidal model was used only as curve fitting as both units from (x,y) were in the form of percentages, similar to the analysis done by Li et al.^51^. Based on Fig. 7, anticancer activity would reach a plateau point if antioxidant activity reaches near 100%, whilst both bioactivities of the extract could reach the 100% point simultaneously only when against HT-29. When against T47D and HeLa, anticancer activity would only slowly increase to almost 50% and 75% respectively despite 100% antioxidant activity. This could mean that at least more than half of the phytochemical compounds present could be attributed to both anticancer and antioxidant activity of the extract. By adding extract concentration, at first the rate of anticancer and antioxidant activity would certainly increase sharply, but the activities would then stagnate towards the point of saturation.

It was also suspected that another possible mechanism of the extract would be similar to the mechanism of the substrate towards enzymes as previously proposed for natural growth factor and HT-29 anticancer activity, in which the concentration of extract would factor significantly by increasing biological activity and will then reach a point of saturation depending on the cancer cell (Table 5). Despite possible anticancer activity stagnation, cancer treatment would also be backed by natural growth factors and anti-aging activity for normal cells due to its antioxidant activity should it be used in vivo or tested in a cancer niche. As the extract would not only become more cytotoxic against cancer cells and better at recognizing cancer cells by antioxidants, it would also help newer normal cells proliferate more and grow with increasing concentration. However, further studies still need to be conducted on cancer stem cells to avoid growing normal and stem cells that would be susceptible to carcinogenesis and drug resistance.

**Table 5:** Hill equation on correlation between antioxidant activity and cell anticancer activity of extract towards cancer cell lines.

Hill equation sigmoidal fitting		
HT-29	HeLa	T47D
$y=101.95\frac{{x}^{4.74}}{{46}^{4.74}+{x}^{4.74}} (Eq.1)$	$y=55.37\frac{{x}^{7.11}}{{54.95}^{7.11}+{x}^{7.11}} (Eq.2)$	$y=74.76\frac{{x}^{5.25}}{{48.99}^{5.25}+{x}^{5.25}} (Eq.3)$
R^2^ = 0.9821	R^2^ = 0.9622	R^2^ = 0.9785

By using 658 ppm of extract, 100% of antioxidant activity might be reached simultaneously with cell enhancement activator at 109% while anticancer activity towards HT-29 is at 100%, 55% for HeLa, and 73% for T47D. The results showed that *X. granatum* leaves ethyl acetate extract could become a new potential for naturally-occurring local anticancer drug for colorectal cancers. As the majority of phytochemicals in the ethyl acetate extract were the antioxidant phenolic compounds, it was suggested that flavonoids might have been the main inhibitors of cancer proliferation. In many cases, antioxidant and cytotoxicity of phenolic compounds work in tandem, for example: several flavonoids such as tangeretin, fisetin, and scutellarin are anti-proliferates; while some other phenolic compounds such as procyanidin b1 and polyphenol gossypol are also known to reduce ROS in cancer cells and may induce apoptosis pathways^6,21,48^. However, as the extract composition was still considered to be very crude, a fractionized version of ethyl acetate extract was tested for cytotoxicity against HT-29.

### Anticancer activity of fractionized *X. granatum* leaves ethyl acetate extract against HT-29

Based on Table 6, all seven fractions had relatively higher MTT anticancer IC_50_ when compared to crude extract MTT IC_50_, except for fractions 5 and 6 with 23.12 and 34.02 ppm respectively while the highest IC_50_ was reached by fraction 4. This would mean that the compounds that correspond to anticancer activity against HT-29 are either semi-polar or relatively polar. Flavonoids were categorized into nonpolar, semi-polar, and polar depending on its structure; with flavones and flavonols easily extracted using either chloroform or dichloromethane. Both of these flavonoidic categories could be regarded as an antioxidative compound as both generalized structures had either ketones or alcohols used to scavenge ROS^6,31,34^. It was also reported that most phenolic phytochemical compounds could be easily extracted with a semi-polar to very polar solvents^52^.

**Table 6:** Anticancer IC_50_ of fractionized extract against colorectal cancer cell (HT-29).

Fractionized extract sample	Anticancer activity IC_50_ (ppm) against HT-29
Extract fraction 1	74.84
Extract fraction 2	80.36
Extract fraction 3	84.05
Extract fraction 4	235.78
Extract fraction 5	23.12
Extract fraction 6	34.02
Extract fraction 7	56.54
Crude extract reference	42.50
CDDP reference	115.91

However, all of the fractions need further characterization and purification into single-compound extracts to elucidate exactly what compounds are contained within and how their individual structures interact with HT-29. From Fig. 1b, it was indicated that the phytochemicals in extract fraction 1 until 4 consisted of blue-banded flavonoids and red-banded anthocyanidins except fraction 1; while the full structure of each phenolic compounds were not known. This might have meant that the most non-polar flavonoids had relatively lower anticancer activity compared to the flavonoids found in other fractions. However, due to the lack of further characterization on the composition of phytochemicals, it was still not possible to determine whether the high potency of anticancer activity in most extracts such as fraction 5 and 6 to contain fully individual flavonoids or some other specific phytochemicals that had been degraded. It was hypothesized that most phytochemicals that had were contained within the extracts had decomposed into simpler forms, thus stability tests on the anticancer activity of extracts were performed.

### Stability of *X. granatum* leaves ethyl acetate extract

While still an incomplete fingerprinting for phytochemicals, Fig. 1b and the phytochemical tests had indicated that the compounds found in *X. granatum* leaves ethyl acetate extract were not structurally stable and might have degraded into simpler phytochemicals that still retain antioxidant as well as growth factor activity. It has been studied that most phenolic compounds are sensitive towards their environment especially by storage conditions such as temperature, light, humidity, and atmosphere^53^; which may indicate how biological activity such as anticancer activity of the extracts could change after stored for some time.

Table 7 showed the comparison between the results of anticancer inhibition activity of freshly prepared *X. granatum* leaves ethyl acetate extract (Table 1) and the results of anticancer inhibition activity of extracts that had been stored for approximately six months and used several times as parent extract for anticancer efficacy tests (Table 4) which were done after toxicity tests. Based on this comparison, the freshly prepared extracts had a significantly higher inhibition percentage than the stored ones, where anticancer inhibition decreased around 19% for HeLa and 10% for the breast cancers. The results showed that storage conditions could affect stability as well as anticancer activity of extracts, with antioxidant activity found in the extracts might be slightly non-optimal^54^.

**Table 7:** Comparison between anticancer inhibition at 500 ppm of freshly prepared and stored *X. granatum* leaves extract against HeLa and breast cancer cells (MCF-7 and T47D).

Extract sample	Anticancer activity, inhibition at 500 ppm (%)	
	HeLa	MCF-7
Cold-stored extract	73.07	86.46(T47D)
Fresh extract	92.96	96.95

Table 7 may be used as a simple stability test to evaluate whether storage conditions could factor the stability of phytochemicals and their SAR as well as their inherent biological activities. However, the results taken should be analyzed qualitatively and only as supporting data and references, as the result could not quantitatively answer how far the phenolics could degrade and that both breast cancer cells MCF-7 and T47D were not the same. Both cells originate from the same cancer subtype luminal A breast cancer, with high similarity between both cells. The difference between both cells could be found in their metabolic receptors, where T47D receptors are susceptible towards both estrogens and progesterone, while MCF-7 only receive from estrogens^55^. Both cells also show diverse protein expressions and different bioenergetics signals, especially for T47D which expressed more proteins involved in cell growth, cancerogenesis, and regulation for cancer apoptosis suppression^56–58^.

As phenolic compounds as well as other unchecked phytochemical compounds could further degrade, storage encapsulation and drug delivery systems are proposed to be researched for extract delivery or for increasing stability and shelf-life of the extracts^1,40^. Ingestion of compounds might not be the best way to deliver the drug as it would lead to digestion and degradation by pH^53^. Nanoparticles however could be utilized as carriers for the extracts if it indeed had high cytotoxicity which could shield the blood vessels where particles move. An example could be in the form of a lipid derivative liposome with high antioxidant extract and modified with cancer-detecting receptors^59^. The liposome is biodegradable and could hold extracts inside by utilizing hydrophobicity of extract, while ingestion or injection to bloodstream could be done without any risks of destroyed nanoparticle carrier. It was also reported that a simple spray-drying and cyclodextrin coating could stabilize the antioxidant activity of phenolic compounds, which might be ideal for increasing shelf-life and biocompatibility with the human body^54^.

### Biological activity of *X. granatum* leaves ethyl acetate extract in terms of ethnogeography

Aside from Indonesia, *X. granatum* has also been found and studied in various tropical and coastal countries like Bangladesh^21^, Thailand^60^, and China^11^. Their geographical features^12^ and ecosystems^61,62^ influenced the diversity of each phytochemical composition as well as their biological activity. Because of this, antioxidant and anticancer activity from wild-type Indonesian *X. granatum* leaves were compared with the results from Indian and Chinese *X. granatum* leaves. Antioxidant activity of local species extract was compared with results from species originated from the Odisha coast of India^12^ in terms of DPPH assay antioxidant IC_50_. It was found out (Table 8) that the antioxidant activity of the leaves extract from local species *X. granatum* had a slightly significant stronger free radical inhibition when compared with the Indian-based species extract (0.01 < p-value < 0.05).

**Table 8:** Comparison between DPPH antioxidant IC_50_ of local and Indian-based crude *X. granatum* leaves extract^12^.

Extract sample source	Antioxidant activity DPPH IC_50_ (ppm)
Local crude leaf extract	84.93 ± 12.93
Indian-based crude leaf extract^12^	110.40 ± 1.56

The local mangrove extract was more active in terms of antioxidants despite the Indian mangrove sample having more variation of phytochemical compounds (terpenoids, glycosides, tannins, flavonoids, and phenols) than the local *X. granatum* leaves ethyl acetate extract. Antioxidant secondary metabolites are closely linked with salinity stress as stress-tolerating plants such as mangroves need to produce compounds that can eliminate free radicals that can form destructive reactions in the plants^63^. Most of the mangroves in India used in the research^12^ had been living in the delta formations near the Indian Ocean, giving rise to a highly stress-tolerant type of *X. granatum*. Mangroves found in the local area (Lampung Mangrove Center) were more stress-tolerant and had better geographical factors than the ones grown in India as the coastal regions of local mangroves were very near to an estuary and were on the shorelines. However, this does not mean that metabolites with both antioxidant or only anticancer activity of stress-tolerant plants were significantly dependent on salinity, as anticancer phytochemical compounds are more related to the defense mechanism of plants against microbes, viral infections, and tumor growth from viral infections^64^.

Anticancer activity of locally grown *X. granatum* leaves ethyl acetate extract was compared qualitatively with the same species originating from the seashores in Hainan Province (Dongzhai Port Nature Reserve) of China^11^, with the Chinese mangrove extract being fractionized into a single compound and assayed using Sulforhodamine B (SRB) against A459 lung cancer cell line, while local species were assayed using MTT against HT-29, T47D, and HeLa cells.

Almost all isolated protolimonoids showed inactive inhibition or cytotoxic activity towards A549 except one at 54.2% for concentration 10 µM, which was Xylogranatumine F (C_36_H_58_O_7_) at 6.028 ppm shown in Table 9^11^. Based on the table, Xylogranatumine F had a stronger cytotoxic effect against A549 when compared with the local species crude extract towards several cancer cell lines, with the highest inhibition percentage reached against HeLa. Despite this, the Xylogranatumine F results were just used as a reference in this article to give some insight on how different biological activity of Indonesian *X. granatum* mangrove extracts when compared to *X. granatum* from outside of Indonesia as well as the significance of fractionized and crude extract in terms of biological activity efficacy.

**Table 9:** Comparison between anticancer inhibition at 6.028 ppm of local *X. granatum* leaves ethyl acetate extract and Chinese-based fractionized compound Xylogranatumine F against cancer cells^11^.

Sample extract source	Anticancer activity, inhibition at 6.028 ppm (%)			
	HeLa	T47D	HT29	A549
Local leaves ethyl acetate crude extract	24.65	2.73	18.54	n.d
Xylogranatumine F from fractionized leaf extract^11^	n.a	n.a	n.a	54.2

*n.a.* not available, *n.d.* not determined.

One reason why the anticancer activity between both extracts differed had to do with the amount of phytochemicals inside the extracts. Single compound extracts are more potent activity than the unrefined crude ones, but dosage-wise crude extract could have a larger activity with larger concentrations^65^, hence why the concentration of purified extract used in the Chinese mangrove results were minuscule. In principle, crude and even fractionized extracts are non-homogenous and mixtures of differing phytochemicals are highly probable. This could lead to some phytochemical compounds interacting with one another in either synergistic or antagonistic behavior^66^. For instance: antimicrobial extracts exhibited synergistic interaction when made from mixing *Filipendula vulgaris* leaf essential oil with salicylaldehyde and linalool; while antagonistic activity was reported when essential oil was mixed with salicylaldehyde and methyl salicylate^67^. It is possible that the anticancerous phytochemicals found in the crude ethyl acetate extract were antagonistic in nature hence, attenuation of anticancer activity. One study found that crude extract made from *Xylocarpus moluccensis* had lower cytotoxicity activity against HepG2 cell line when compared to diethyl ether extract due to some unreported compounds produced antagonistic interactions^68^.

The local crude extract had a composition of many unknown phytochemical compounds as well as metabolic byproducts which might or might not have been degraded. This led to antagonism on the anticancer activity and to some extent antioxidant activity of the compounds, thus attenuating cancer cells inhibition and decreasing potency compared to C_36_H_58_O_7_ against A549^11^. Due to this, further research in metabolomics on the crude extract is essential to determine which compound releases what activity and how the compounds affect the whole crude extract system in terms of SAR. Pharmacokinetics could also be done to determine what phytochemical mixtures could hypothetically be used to synergize the already crude extract or a fractionated version of the extract.

To summarize, phytochemicals from local mangrove species *X. granatum* leaves ethyl acetate solvent crude extract was characterized albeit still incomplete due to various problems such as time constraints. It was suggested that flavonoids and some other phenolic elements were found to be the major contributor of antioxidant activity phytochemical compound. These phenolic compounds were suggested to exhibit intermediate antioxidant activity as well as a degree of anticancer activity, which was found to be most effective against HT-29 with anticancer IC_50_ of one fractionized extract reaching approximately 20 ppm against HT-29. The extracts also behaved as a natural growth factor and anti-aging due to its antioxidant activity towards brine shrimps and hADSC. The stability test also showed that phenolic compounds in extract had slightly degraded due to storage factors.

The ethyl acetate extracts were also compared with a couple of studies for the same species and plant part in terms of different ethnogeographical site for biological activities, with local species of *X. granatum* leaves ethyl acetate crude extract having stronger antioxidant activity than the Indian mangrove species and inconclusive results when compared with Chinese mangrove species for anticancer activity due to local extracts not purified into single-compound.

It was suggested for further research on extract purification, SAR and apoptosis mechanism, drug delivery and encapsulation system, and pharmacokinetics/pharmacodynamics of extract to determine whether the extract could be used in potential anticancer-antioxidant drug.

## Methods

All experimental research and field studies on plants, including the collection of plant material in this study, had complied with relevant institutional, national, and international guidelines and legislation.

### Leaves simplicia extraction and solvent determination

The identification of plant materials used in this study were conducted by Dr. Joeni Setijo Rahajoe at the Herbarium of the Indonesian Institute of Sciences, Biology Research Center as reported in our prevous publication^16^, with identification number 1570/IPH.1.01/If.07/VI/2017. The plant materials were collected with the permission, and guidance by the local authority at Kawasan Konservasi Mangrove, Lampung Province, Indonesia. An official letter of permission was submitted and approved by the authorities prior to sample collection.

Simplicia leaves of *X. granatum* from Kawasan Konservasi Mangrove, Lampung Province, Indonesia (GPS Coordinates of Samples: -5.545061, 105.770815) were extracted by means of maceration and evaporation using locally-bought solvents with different polarity: ethyl acetate, ethanol, and water. Ethyl acetate was chosen as the solvent for extraction based on its cancer growth inhibition quality and as more compatible for extracting phytochemicals contained in the *X. granatum* leaves due to their chemical and structural properties^14^.

### Extract fingerprinting by TLC and fractionation

*X. granatum* simplicia leaves extracted in ethyl acetate were dissolved in methanol and placed on TLC plates Silica G50F254 after the plates had been eluted by either methanol, ethyl acetate, dichloromethane, chloroform, acetone, ethanol, or n-hexane. Color bands were observed in UV-light at 254 and 366 nm. Chloroform:dichloromethane made with ratio 9:1 (v/v) was chosen as chromatography eluent as it was compatible with fractionation of semi-polar phytochemicals in extract^16^. Fractionation solvent were to be further used for TLC extract fingerprinting and column chromatography.

Extracts were then fractionated by isocratic method with Silica60 as stationary phase and chloroform:dichloromethane (9:1) as mobile phase. Fractionation was done in seven parts and visualized in a TLC plate to determine fraction amount. Extract was also fractionized by column chromatography by separating the phytochemicals depending on their polarity, with nonpolar chemicals being eluted first by mobile phase chloroform:dichloromethane (9:1). The purified extracts were then assayed for cytotoxicity against cancer and normal cells.

### ^1^H NMR spectroscopy for extract compound characterization

Six replicates of *X. granatum* leaves ethyl acetate extracts were mixed with CD_3_OD and D_2_O dissolved in KH_2_PO_4_ buffer solution pH 6.0 containing 0.1% trimethylsilylpropanoic acid (TSP). Homogenized mixtures were centrifuged and supernatants were taken to NMR tube for spectral analysis using 500 MHz NMR spectrometer Varian Inova 500 (Varian, USA) at 25 °C, functioning at frequency 449.91 MHz. The acquisition time for each 1H NMR spectrum was 3.53 min, consisting of 64 scans with a width of 12 ppm. Phase, baseline corrections of spectra and TSP calibrations for chemical shift indicators were conducted using software Chenomx version 8.1. Additional support for identification was obtained using two-dimensional (2D) J-resolved (JRES) NMR spectroscopy. Identification of some metabolites and analysis support for spectral database matching was possible using 1D and 2D NMR by comparison with databases such as Human Metabolome Database (HMDB, http://www.hmdb.ca/), and Biological Magnetic Resonance Data Bank (BMRB, http://www.bmrb.wisc.edu/), aided by Chenomx NMR Suite 7.7 (Chenomx Inc., Edmonton, Canada).

All NMR spectra were manually phased with baseline calibrated to TSP at 0.00 ppm. Chemical shift region at 0 to 10 were reduced to integrated bins of 0.04 ppm width to be used in Chenomx NMR Suite 5.1 Professional for multivariate pattern recognition analysis. The spectral region associated with residual water (4.66–5.05 ppm) was removed. The remaining spectral segments for each NMR spectrum were normalized to the total sum for the spectral intensity to partially compensate for the difference in concentration. NMR data were analyzed and modeled by multivariate statistical methods using SIMCA-P 13.0 software package. The data were mean-centered and Pareto scaled prior to analysis for principal component analysis (PCA) and partial least squares (PLS) regression analysis, with visualization of data taken from score plot of two principal components (PC1 and PC2) representing individual spectrum of a sample. Metabolites associated with group separation were indicated b corresponding plot, in which each point stood for a single NMR spectral bin.

### Quantitative and qualitative phytochemical analysis

Simplicia leaves were phytochemically analyzed qualitatively^13^ by Mayer’s test, Wagner’s test, and Dragendroff’s test for alkaloid detection; froth test for saponin detection; phenol’s Ferric Chloride test for tannin detection, Liebermann Buchard’s test, and Salkowski’s test for steroids and triterpenoid detection. Flavonoid types were detected by Willstatter’s reaction test, while hydroquinones were detected by alkaline reagent test^14^. Quantitative tests were also performed for measuring the phenolic content of extracts such as TPC and FC.

### TPC determination

Method was adapted from Audah et al.^16^ with several modifications in the procedure. Ethyl acetate extract paste was dissolved using 75% ethanol (Merck, Germany). The extracts were mixed with Na_2_CO_3_ 2% (Sigma Aldrich, USA) and locally bought Folin-C. Reagent 10% (1:2:1). The mixtures were incubated for 30 min in darkness at room temperature. Mixtures were then read at 765 nm wavelength using Cary 60 UV light-visible (UV–vis) spectrophotometer (Agilent Technologies, USA). TPC concentration of extracts was calculated using a standard calibration curve made from gallic acid (Sigma Aldrich, USA) with the same procedure at 0–100 ppm.

### FC determination

Method was adapted from Audah et al.^16^ with several modifications in the procedure. Ethyl acetate extract dissolved in 75% ethanol from stock solution used in TPC determination were mixed with 75% ethanol, 20% of locally bought AlCl3, 2 M of CH_3_COOK (BDH Middle East, Dubai), and aquadest (5:15:1:1:28). The mixtures were incubated 30 min in darkness at room temperature and were read at 440 nm UV–vis. The FC concentrations of extracts were calculated using standard quercetin (Sigma Aldrich, USA) at 0–200 ppm.

### Antioxidant activity determination by DPPH radical scavenging assay

Antioxidant assay was performed as reported by Jadid et al.^15^, with several modifications in the procedure. Ethyl acetate extract and ascorbic acid (Merck, Germany) dissolved in 75% ethanol at concentrations 0–100 ppm were mixed with DPPH (Sisco Research Laboratories, India) 100 ppm (1:1). The mixtures were incubated for 30 min in a dark chamber at 25 °C and read at 517 nm UV–vis with both DPPH IC_50_ made from standard control calibration curve DPPH at concentration 0–100 ppm. Both IC_50_ were then compared with each other.

### BSLT for extract toxicity assay

The experiment was performed according to the methods reported by Dosumu et al.^37^, with several modifications in the procedure. Ethyl acetate extracts dissolved with seawater at concentrations 0–1500 ppm in triplicates were mixed with seawater (1:1) filled with 10 *A. salina* nauplii which were hatched 24 h before experiment using CO_2_ aerator. Mixtures were incubated for 24 h at 25 °C and BSLT LC_50_ of extracts were calculated using probit analysis, with a positive standard of 50% ethanol and negative standard of seawater solution with 10 brine shrimps in each solution.

### MTT cytotoxicity assay for cytotoxicity and anticancer activity determination

Liquid mediums for cells were made to prepare cancer cell lines HeLa, MCF-7, T47D, and HT-29 for cancer cell cytotoxicity test as well as stem cell hADSC for normal cell cytotoxicity test. For HeLa, MCF-7, and HT-29, mediums were made from Gibco Dulbecco’s Modified Eagle Medium (DMEM) (Thermo Fisher Scientific, USA), Fetal Bovine Serum (FBS) 10% (BBI Life Sciences, China), and Penicillin–Streptomycin antibiotics mixture (PenStrep) 1% from Life Technologies (USA). Medium for T47D cells were made from Gibco RPMI 1640 (Thermo Fisher Scientific, USA), 10% FBS, and 1% PenStrep. Medium for hADSC was made from DMEM, PenStrep 1%, and platelet-rich plasma serum (PrP) which was made based on the recipe of Suryani et al.^69^. Cells were harvested using Gibco Trypsin/EDTA 0.25% (Thermo Fisher Scientific, USA), with all reagents and chemicals diluted using phosphate saline buffer (PBS) tablets from Biomatik, Canada.

Extracts and Cisplatin drug (CDDP) (Kalbe Farma, Indonesia) were dissolved in a liquid medium made for each cell and dimethyl sulfoxide (DMSO) (Biomatik, Canada) 2% specifically for extract. Solutions were made in triplicate on concentrations 0–1000 ppm for extract and 0–100 ppm for CDDP were incubated into 96-well microtiter plate containing cells harvested at confluence 80% (density 10,000 cell/well after 24 h) for 24 h at 5% CO2 using Heracell 150i CO2 Incubator (Thermo Fisher Scientific, USA). Plates were re-incubated 4 h after the addition of Thiazolyl blue tetrazolium bromide (BBI Life Sciences, China) 500 ppm and were added with DMSO to dissolve any formed formazan crystals. Plates were visualized with Eclipse Ti-S Inverted Microscope (Nikon Instruments, Japan) and scanned with Varioskan LUX Multimode Microplate Reader (Thermo Fisher Scientific, USA) at 595 nm wavelength with anticancer MTT IC50 of CDDP and extracts compared. Control solutions of the plates were made for negative controls using only DMSO 2% as mediums for cells, normal mediums only with cells, normal mediums with no cells for blank solutions, and blank extracts and medium mixtures.

### Statistical analysis

All values were expressed as mean ± standard deviation, with all samples being made at least in triplicates. Statistical analysis was done using Student’s one-tailed t-test for comparison analysis and probit analysis for LC_50_, both performed in Microsoft Excel 2013. Linear regression was also done in Microsoft Excel 2013, with sigmoidal fitting done in OriginPro 2018 Academic (License 95E from KIT institution) using Hill’s model equation as a basis. Statistical significance for hypothesis testing was accepted for *p-*value < 0.05 (slightly significant) and < 0.01 (significant). NMR data was analyzed with model significance visualized by SIMCA-P 13.0 using permutation tests and CV-ANOVA. One-way ANOVA was also done for NMR data in GraphPad Prism ver. 6.0 for data interpretation as Tukey’s test applied as post hoc analysis method; with NMR statistical value expressed as mean ± standard error of mean with *p-*value < 0.05 considered as significantly different.

Statistical analysis using IBM SPSS Statistics for Windows Version 25.0 was used to conduct one-way Analysis of Variance (ANOVA) tests to explore the comparative effects of *X. granatum* extract types—500 ppm samples of Water, Ethanol, Ethyl Acetate and DOX as a Drug Control—for cytotoxicity (%Inhibition as dependent variable) on MCF-7 and HeLa cancer cell types. Furthermore, the software was used to observe the comparative effects of Sample types on cancer activity (IC_50_ as dependent variable)—*X. granatum* leaves ethyl acetate extract or CDDP samples across hADSC, HeLa, T47D, and HT-29 cell types. Brown-Forsythe (BF) test was conducted to determine the equality of each group variance on ANOVA after assumptions on Levene’s Test for Homogeneity of Variance had been violated. Post hoc tests on comparative effects of *X. granatum* extract types as well as *X. granatum* leaves ethyl acetate and CDDP samples across different cell lines using Games-Howell test to explore each group’s comparative effects.

## Supplementary Information

Supplementary Information 1.

Supplementary Information 2.

## Data Availability

Data and sample materials used in the research are available from the corresponding author.
