# Supplementary material for: Evaluation of Indonesian mangrove Xylocarpus granatum leaves ethyl acetate extract as potential anticancer drug
Source: Sci Rep. 2021 Mar 16;11:6080. doi: 10.1038/s41598-021-85383-3 (PMC7971038; doi:10.1038/s41598-021-85383-3)
Supplement: Supplementary file 1 — Supplementary Information 1. [file 41598_2021_85383_MOESM1_ESM.pdf]

Evaluation of Indonesian Mangrove *Xylocarpus granatum* Leaves Ethyl Acetate Extract as Potential Anticancer

Jason Darmadi[a], Razethy Rahayu Batubara[a], Sandiego Himawan[a], Norma Nur Azizah[b], Hilyatushalihah Kholis Audah[a], Ade Arsianti[b,c], Evi Kurniawaty[d], Intan Safinar Ismail[e], Irmanida Batubara[f,g], Kholis Abdurachim Audah[a,h,*]

a. Department of Biomedical Engineering, Swiss German University, 15143 Tangerang, Indonesia
b. Drug Development Research Center, IMERI, University of Indonesia, 10430 Jakarta, Indonesia
c. Department of Medical Chemistry, University of Indonesia, 10430 Jakarta, Indonesia
d. Faculty of Medicine, University of Lampung, 35145 Bandar Lampung, Indonesia
e. Institute of Bioscience, Universiti Putra Malaysia, 43400 Serdang, Malaysia
f. Biopharmaca Tropica Research Center, IPB University, 16680 Bogor, Indonesia
g. Department of Chemistry, IPB University, 16680 Bogor, Indonesia
h. Directorate of Academic Research and Community Service, Swiss German University, 15143 Tangerang, Indonesia

* Corresponding Author: kholis.audah@sgu.ac.id; Tel.: +62-212-9779-596/9-598

Supplementary Materials:

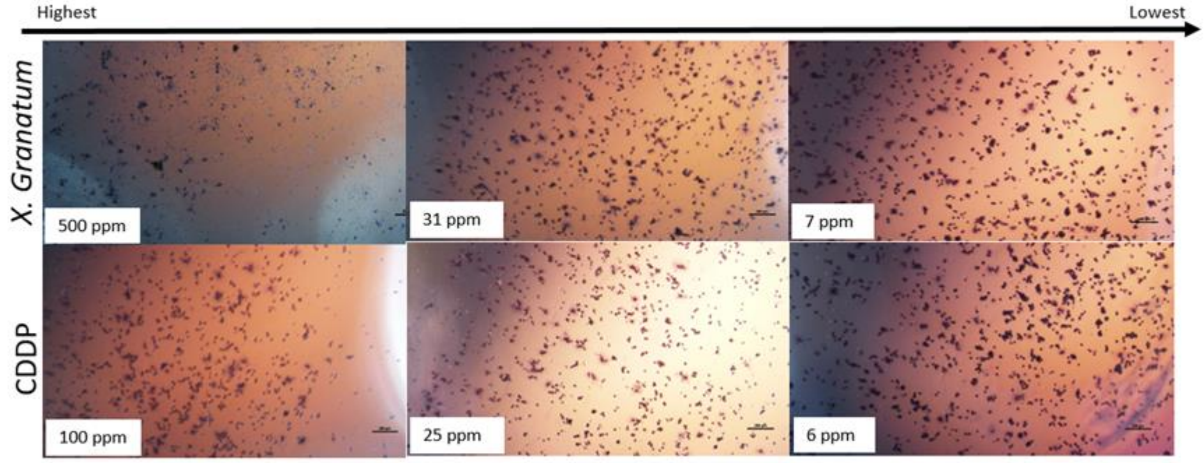

Figure S1. Comparison between colorectal cancer HT-29 cell line after being exposed to reference drug Cisplatin (CDDP) and *Xylocarpus granatum* leaves ethyl acetate crude extract at different concentrations.

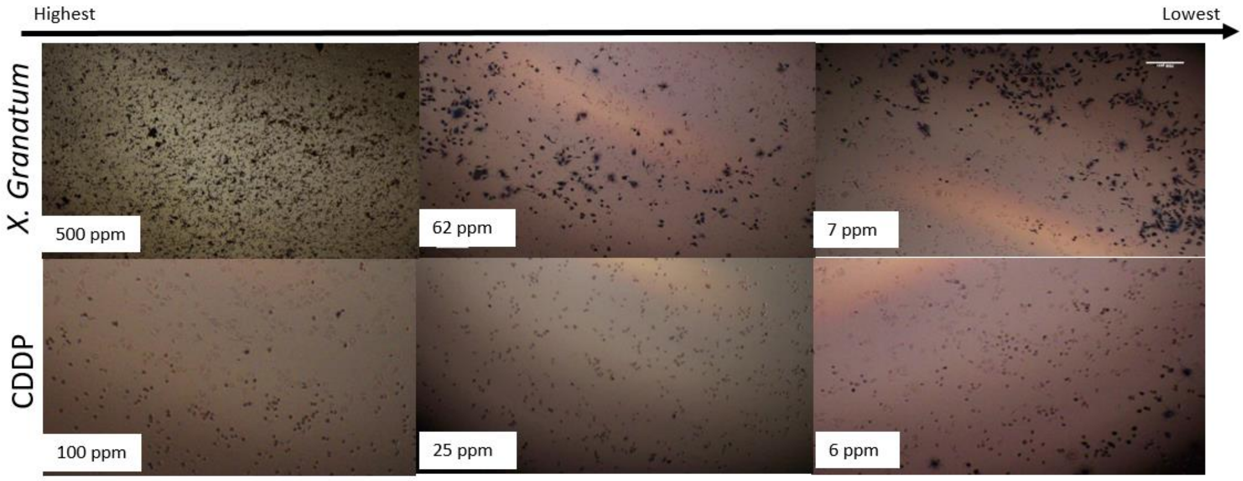

Figure S2. Comparison between cervical cancer HeLa cell line after being exposed to reference drug CDDP and *X. granatum* leaves ethyl acetate crude extract at different concentrations.

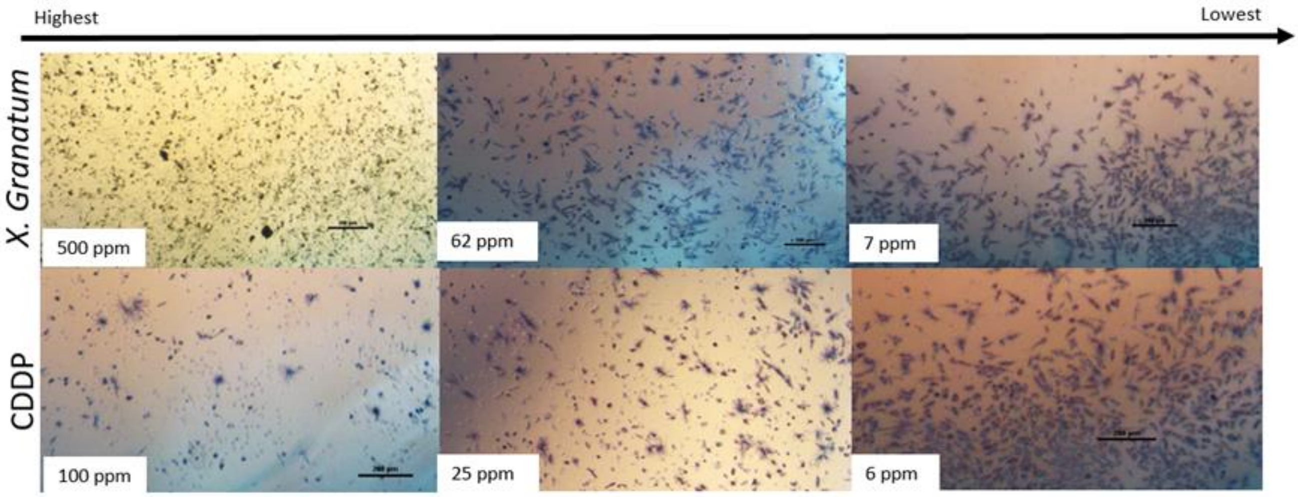

Figure S3. Comparison between breast cancer T47D cell line after being exposed to reference drug CDDP and *X. granatum* leaves ethyl acetate crude extract at different concentrations.

Table S1. Correlation between antioxidant activity of *X. granatum* leaves ethyl acetate crude extract with its anticancer activity as well as its stem cell enhancement activator effect.

Concentration (ppm)	Antioxidant Inhibition (%)	Anticancer Inhibition (%)			Cell Enhancement Activator (%)
		HT-29	HeLa	T47D	
0	42,78	-	-	-	-
6,25	43,32	18,63	-19,88	2,72	19,32
12,5	43,86	30,74	-8,65	13,27	32,66
25	44,93	42,85	2,58	23,82	46,01
50	47,09	54,96	13,81	34,37	59,35
70,7	48,87	61,01	19,42	39,64	66,02
100	51,4	67,06	25,04	44,92	72,69
170	57,43	76,33	33,64	53,00	82,91
200	60,02	79,17	36,27	55,47	86,03
250	64,33	83,07	39,89	58,87	90,33
400	77,26	91,28	47,50	66,02	99,38
500	85,88	95,18	51,12	69,42	103,67
658.6	99,55	99,99	55,58	73,61	108,97
800	111,74	103,39	58,73	76,57	112,72
1000	128,98	107,29	62,35	79,97	117,01
1600	180,7	115,50	69,96	87,12	126,06
1800	197,94	117,56	71,87	88,91	128,33
2000	215,18	119,40	73,58	90,52	130,36
2500	258,28	123,30	77,19	93,91	134,65
3000	301,38	126,48	80,15	96,69	138,16
4000	387,58	131,51	84,81	101,07	143,70
8000	732,38	143,62	96,04	111,62	157,04
10000	904,78	147,51	99,65	115,01	161,34

Table S2. Results of the Shapiro-Wilk Statistical test of normality for comparative analysis of *X. granatum* Leaves Water extract, Ethanol extract, Ethyl Acetate Extract and Doxorubicin drug control with their anticancer activity.

		Shapiro-Wilk		
	**Extract Type**	**Statistic**	**df**	**Sig.**
**MCF-7**	Water	.903	3	.396
	Ethanol	.939	3	.525
	Ethyl Acetate	.978	3	.715
	Dox	.937	3	.514
**HeLa**	Water	.903	3	.396
	Ethanol	.913	3	.427
	Ethyl Acetate	.750	3	.000
	Dox	.994	3	.849

Table S3. Results of the Levene's test of Homogeneity of variances for the comparative analysis of *X. granatum* Leaves Water extract, Ethanol extract, Ethyl Acetate Extract and Doxorubicin drug control with their anticancer activity.

		**Levene Statistic**	**df1**	**df2**	**Sig.**
**MCF-7**	Based on Mean	7.977	3	8	.009
	Based on Median	1.446	3	8	.300
	Based on Median and with adjusted df	1.446	3	2.303	.416
	Based on trimmed mean	7.114	3	8	.012
**HeLa**	Based on Mean	9.279	3	8	.006
	Based on Median	1.657	3	8	.252
	Based on Median and with adjusted df	1.657	3	2.230	.383
	Based on trimmed mean	8.257	3	8	.008

Table S4. Results of the Oneway ANOVA F test for the comparative analysis of *X. granatum* Leaves Water extract, Ethanol extract, Ethyl Acetate Extract and Doxorubicin drug control with their anticancer activity.

**ANOVA**						
		**Sum of Squares**	**df**	**Mean Square**	**F**	**Sig.**
**MCF-7**	Between Groups	5336.422	3	1778.807	71.884	.000
	Within Groups	197.965	8	24.746		
	Total	5534.387	11			
**HeLa**	Between Groups	1757.331	3	585.777	12.571	.002
	Within Groups	372.778	8	46.597		
	Total	2130.109	11			

Table S5. Results of the Brown-Forsythe F test for the comparative analysis of *X. granatum* Leaves Water extract, Ethanol extract, Ethyl Acetate Extract and Doxorubicin drug control with their anticancer activity.

Robust Tests of Equality of Means					
		**Statistic**[a]	**df1**	**df2**	**Sig.**
**MCF-7**	Brown-Forsythe	71.884	3	2.363	.007
**HeLa**	Brown-Forsythe	12.571	3	2.242	.060

a. Asymptotically F distributed.

Table S6. Results of the Games-Howell Post-hoc analysis for the comparative analysis with multiple comparisons of *X. granatum* Leaves Water extract, Ethanol extract, Ethyl Acetate Extract and Doxorubicin drug control with their anticancer activity.

Multiple Comparisons								
**Dependent Variable**		**(I) Extract Type**	**(J) Extract Type**	**Mean Difference (I-J)**	**Std. Error**	**Sig.**	**95% Confidence Interval**	
							**Lower Bound**	**Upper Bound**
**MCF-7**	**Games-Howell**	Water	Ethanol	-30.9066667	5.7076654	.062	-65.061399	3.248065
			Ethyl Acetate	-52.8233333*	5.5087224	.026	-90.749909	-14.896758
			Dox	-50.1266667*	5.5310256	.028	-87.536990	-12.716344
		Ethanol	Water	30.9066667	5.7076654	.062	-3.248065	65.061399
			Ethyl Acetate	-21.9166667*	1.5497885	.010	-31.864291	-11.969042
			Dox	-19.2200000*	1.6272881	.008	-28.082876	-10.357124
		Ethyl Acetate	Water	52.8233333*	5.5087224	.026	14.896758	90.749909
			Ethanol	21.9166667*	1.5497885	.010	11.969042	31.864291

			Dox	2.6966667	.6454800	.074	-.444188	5.837521
		Dox	Water	50.1266667*	5.5310256	.028	12.716344	87.536990
			Ethanol	19.2200000*	1.6272881	.008	10.357124	28.082876
			Ethyl Acetate	-2.6966667	.6454800	.074	-5.837521	.444188
**HeLa**	**Games-Howell**	Water	Ethanol	-21.0600000	7.8771209	.251	-69.596352	27.476352
			Ethyl Acetate	-28.1100000	7.6543648	.160	-81.131492	24.911492
			Dox	-30.9033333	7.6589867	.136	-83.813412	22.006746
		Ethanol	Water	21.0600000	7.8771209	.251	-27.476352	69.596352
			Ethyl Acetate	-7.0500000	1.8626683	.152	-19.914228	5.814228
			Dox	-9.8433333	1.8815714	.079	-22.283010	2.596344
		Ethyl Acetate	Water	28.1100000	7.6543648	.160	-24.911492	81.131492
			Ethanol	7.0500000	1.8626683	.152	-5.814228	19.914228
			Dox	-2.7933333*	.2838623	.018	-4.525592	-1.061075
		Dox	Water	30.9033333	7.6589867	.136	-22.006746	83.813412
			Ethanol	9.8433333	1.8815714	.079	-2.596344	22.283010

	Ethyl Acetate	2.7933333*	.2838623	.018	1.061075	4.525592

*. The mean difference is significant at the 0.05 level.

Table S7. Results of the Shapiro-Wilk Statistical test of normality for *X. granatum* and CDDP IC50 samples across cell types.

	Cell-type	Shapiro-Wilk		
		**Statistic**	**df**	**Sig.**
**IC50_X. granatum**	hADSC	.802	3	.119
	HeLA	.809	3	.136
	T47D	.872	3	.302
	HT-29	.902	3	.392
**IC50_CDDP**	hADSC	.840	3	.215
	HeLA	.750	3	.001
	T47D	.954	3	.587
	HT-29	.999	3	.927

Table S8. Results of the Levene's test of homogeneity for *X. granatum* and CDDP IC50 samples with their cytotoxic activity.

		**Levene Statistic**	**df1**	**df2**	**Sig.**
**IC50_X. granatum**	Based on Mean	13.971	3	8	.002
	Based on Median	1.168	3	8	.380
	Based on Median and with adjusted df	1.168	3	2.025	.491
	Based on trimmed mean	11.410	3	8	.003
**IC50_CDDP**	Based on Mean	15.658	3	8	.001
	Based on Median	.977	3	8	.450
	Based on Median and with adjusted df	.977	3	2.001	.542
	Based on trimmed mean	12.337	3	8	.002

Table S9. Results of the Oneway ANOVA F test used to observe the differences between *X. granatum* and CDDP IC50 samples with their cytotoxic activity.

ANOVA						
		**Sum of Squares**	**df**	**Mean Square**	**F**	**Sig.**
**IC50_X. granatum**	Between Groups	607577.460	3	202525.820	1.092	.406
	Within Groups	1483217.592	8	185402.199		
	Total	2090795.052	11			
**IC50_CDDP**	Between Groups	2893781.236	3	964593.745	.931	.469
	Within Groups	8290515.784	8	1036314.473		
	Total	11184297.020	11			

Table S10. Results of the Brown-Forsythe F test used to observe the differences between *X. granatum* and CDDP IC50 samples with their cytotoxic activity.

Robust Tests of Equality of Means					
		**Statistic**[a]	**df1**	**df2**	**Sig.**
**IC50_X. granatum**	Brown-Forsythe	1.092	3	2.032	.509
**IC50_CDDP**	Brown-Forsythe	.931	3	2.003	.555

a. Asymptotically F distributed.
